# Novel Thin Film Nanocomposite Membranes Based on Chitosan Succinate Modified with Fe-BTC for Enhanced Pervaporation Dehydration of Isopropanol

**DOI:** 10.3390/membranes12070653

**Published:** 2022-06-25

**Authors:** Katsiaryna Burts, Tatiana Plisko, Mariia Dmitrenko, Andrey Zolotarev, Anna Kuzminova, Alexandr Bildyukevich, Sergey Ermakov, Anastasia Penkova

**Affiliations:** 1 Institute of Physical Organic Chemistry, National Academy of Sciences of Belarus, 220072 Minsk, Belarus; katyaburt@gmail.com (K.B.); uf@ifoch.bas-net.by (A.B.); 2 Department of Analytical Chemistry, Institute of Chemistry, St. Petersburg State University, 7/9 Universitetskaya nab., 199034 St. Petersburg, Russia; m.dmitrienko@spbu.ru (M.D.); andrey.zolotarev@spbu.ru (A.Z.); a.kuzminova@spbu.ru (A.K.); s.ermakov@spbu.ru (S.E.); a.penkova@spbu.ru (A.P.)

**Keywords:** chitosan succinate, thin film nanocomposite membrane, dynamic technique, physical adsorption, metal-organic frameworks, Fe-BTC, pervaporation, isopropanol dehydration

## Abstract

The application of environmentally friendly and energy-efficient membrane processes allows improvement the ecological safety and sustainability of industrial production. However, the effective application of membrane processes requires novel high-performance thin film composite (TFC) membranes based on biopolymers to solve environmental problems. In this work for the first time novel thin film nanocomposite (TFN) membranes based on biopolymer chitosan succinate (ChS) modified with the metal organic framework iron 1,3,5-benzenetricarboxylate (Fe-BTC) were developed for enhanced pervaporation dehydration. The formation of a selective layer of TFN membranes on the porous membrane-support was carried out by two methods—dynamic technique and physical adsorption. The effect of the membrane formation method and Fe-BTC content in ChS layer on the structure and physicochemical properties of TFN membranes was investigated. The developed TFN ChS-based membranes were evaluated in the pervaporation dehydration of isopropanol (12–30 wt.% water). It was found that TFN ChS-Fe-BTC membranes prepared by two methods demonstrated improved permeation flux compared to the reference TFC ChS membrane. The best transport properties in pervaporation dehydration of isopropanol (12–30 wt.% water) were possessed by TFN membranes with 40 wt.% Fe-BTC prepared by dynamic technique (permeation flux 99–499 g m^−2^ h^−1^ and 99.99% water in permeate) and TFN membranes with 5 wt.% Fe-BTC developed by physical adsorption (permeation flux 180–701 g m^−2^ h^−1^ and 99.99% water in permeate).

## 1. Introduction

In recent years, requirements for the quality, purity, and environmental friendliness of produced substances have been constantly increasing [1]. Therefore, the development of novel energy-efficient and eco-friendly synthesis and separation methods for various compounds, as well as purification technologies, are becoming urgently needed [2,3,4]. Membrane technologies meet all the requirements of “sustainable processes” and are of great interest as an alternative to traditional separation methods due to their advantages: cost- and energy-effectiveness, ecological safety, waste-free, and the ease of application and automation [5,6,7,8]. Pervaporation is one of the most promising membrane methods for the separation of the liquid mixtures of low molecular weight substances, especially for the thermally unstable and close-boiling compounds, azeotropic and isomeric mixtures [9]. The main application of this method is focused on dehydration purposes (selective removal of water from other components, in particular alcohol and solvents) [10].

Alcohols are important chemicals used in various industries (medicine, pharmaceutical, food, chemical, etc.) and as a promising alternative fuel for automobiles, where extreme purity is essential. An isopropanol (iPrOH)/water mixture is often investigated as a model system for pervaporation dehydration, as iPrOH is an industrial solvent actively used instead of ethanol in cosmetics, perfumes, disinfectants, windshield washers, antifreeze liquids and medicine [11]. This mixture is also difficult to separate by traditional methods (rectification and distillation), since it contains an azeotrope with a water content of 12 wt.% and a boiling point of 80.3 °C [12]. It is necessary to add harmful organic intermediates that form stronger azeotropic mixtures with water, which contribute to an additional stage of alcohol purification. In addition, these traditional separation methods are cost- and energy-consuming. Therefore, a promising method for dehydrating isopropanol is pervaporation, which makes it possible and easy to extract water without any additional chemical reagents by the right choice of membrane based on hydrophilic polymers. Polyvinyl alcohol (PVA) [11,13], sodium alginate (SA) [9,14,15,16,17], polyvinyl amine [18], chitosan [19,20,21], poly(ionic liquid) complex (PILC) [22], etc. have been already investigated as a membrane materials for pervaporation dehydration of isopropanol.

The rapid development of industrial technologies focused on environmental protection leads to the need for developing novel pervaporation membranes based on biopolymers. Chitosan is the most abundant natural biopolymer that has many applications in pharmacology, drug delivery, biomedicine, food processing, agriculture, catalysis, and as a membrane material [23]. It is important to note that chitosan membranes, due to their high hydrophilicity, are subjected to predominant swelling in water, resulting in a significant decrease in selectivity because of the plasticization effect [24]. The enhancement of the water swelling resistance of chitosan-based membranes is usually carried out by (i) cross-linking with glutaraldehyde [25] or maleic acid [26], (ii) blending with polyvinyl alcohol [27], poly(N-vinyl-2-pyrrolidone) [28], etc., (iii) introduction of nanoparticles such as titanium dioxide [29], silica [30], graphene oxide [31], carbon nanotubes [32], metal organic frameworks [33], etc., but most often this modification results in a decrease of permeation flux for chitosan membranes. Chitosan can be also converted into various derivatives for specific applications [23]. Chitosan succinate (ChS) is one of the chitosan derivatives and is widely used in the food industry (artificial food products), cosmetics (stabilization of foams, creams, emulsions) and in biotechnology (drug delivery) [34]. It is reported that modification of chitosan membranes with succinic acid in situ during membrane preparation leads to the binding of the carboxyl group (-COO-) of acid to the -NH_2_ group of polymer forming cross-links, which can adjust the distance between polymer chains, forming a neater network structure [35]. This further facilitates component transport through the membrane and the hydrophilicity of the membrane caused by the formation of additional hydrogen bonds between a free -O acid group and water molecules. However, to our knowledge, there is only one work on the development of porous ChS/polyvinyl alcohol-polyethylene glycol membrane which was characterized using creatinine [35], and there is no literature data on the development and investigation of pervaporation ChS membranes. Thus, in this work, for the first time, thin film composite (TFC) membranes based on ChS were developed and studied.

Thin film composite (TFC) membranes are usually obtained by formation of an ultrathin selective polymer layer on an ultra- or micro-filtration membrane-support. Due to the small thickness of the selective layer, they have substantially higher permeation flux compared to dense membranes without deterioration of the selectivity that is promising for industrial application. The preparation advantages of TFC membranes are the possibility to adjust the porosity and nature of a membrane-support, as well as the structure of a membrane selective layer, independently. There are various well-established techniques for selective layer formation on the surface of the porous membrane-support: interfacial polymerization, layer-by-layer deposition, physical adsorption, coating, dynamic technique, etc. Among these, dynamic technique is a promising method of TFC membrane preparation and consists of the filtration of the dilute colloid or polymer solution through the porous micro- or ultrafiltration membrane at a transmembrane pressure difference created by the elevated pressure at the feed side or by reduced pressure on the permeate side. The formation of the thin selective layer in the dynamic technique is based on the phenomena of concentration polarization. The advantages of this technique are: (1) simple implementation of the preparation process; (2) small volumes of required reagents; (3) single-stage process; (4) high speed of preparation without additional reagents; (5) possibility of variation of the properties by changing the selective layer structure and thickness [36,37]. According to Anantharaman et al. [38], the number of reported studies on development of membranes by the dynamic technique has increased. These membranes obtained frequently with polymers, metal oxides, powdered activated carbon, soil, nanoparticles both in cross-flow and dead-end filtration modes, as well as by using reduced pressure on the permeate side of the membrane, applied for micro-, ultra-, nano-filtration, reverse osmosis and pervaporation. Thus, in this work, for the first time, thin film composite (TFC) membranes based on ChS were developed by two methods—physical adsorption and dynamic technique, to assess the influence of the preparation approach on the properties of membranes.

One of the other effective methods to improve membrane performance is the modification of polymer matrix with nanoparticles [39] such as fullerene derivatives [40], carbon nanotubes (CNTs) [41], graphene oxides [42], metals and their oxides [43,44], metal-organic frameworks (MOFs) [45,46], silica [47], etc. Membranes with MOF as a nano-filler have great prospects due to the design simplicity and the possibility of modifying MOFs, as well as the compatibility between MOF and the polymer matrix [48]. Additionally, the MOF introduction into the membrane significantly affects the sorption characteristics, surface hydrophilic-hydrophobic balance, and the free volume of the polymer film due to the MOF porous structure, leading to enhanced transport properties (the increase in the permeation flux and/or selectivity). Fe-BTC is a commercial, unusual MOF representative due to its crystalline/amorphous nature with a microporous structure (window sizes of 5.5 and 8.6 Å) [49,50,51]. Fe-BTC was successfully applied as a modifier for a pervaporation membrane based on sodium alginate for the dehydration of isopropanol (12–100 wt.% water) [52] and polylactic acid for the separation of a methanol/methyl tert-butyl ether (MTBE) mixture [53]. There is no information, to the best of our knowledge, on pervaporation membranes based on ChS modified with Fe-BTC particles.

Thus, in this work for the first time novel thin film nanocomposite (TFN) membranes based on ChS modified with Fe-BTC were developed for enhanced pervaporation dehydration. The effect of the formation method (dynamic technique and physical adsorption) on the membrane structure, topology, hydrophilic-hydrophobic balance and pervaporation performance was studied. The influence of the Fe-BTC concentration in the ChS selective layer on structure, physicochemical properties and pervaporation performance of TFN membranes was investigated. The structural and physicochemical properties of membranes were studied by Fourier transform infrared spectroscopy (FTIR), scanning electron (SEM) and atomic force (AFM) microscopies, and contact angle measurements. Transport characteristics of the TFN ChS-based membranes were evaluated in the pervaporation dehydration of isopropanol (12–30 wt.% water). The best TFN membranes prepared by two techniques were compared and discussed from the point of view of the mechanism of selective layer formation.

## 2. Materials and Methods

### 2.1. Materials

**Figure 1:**
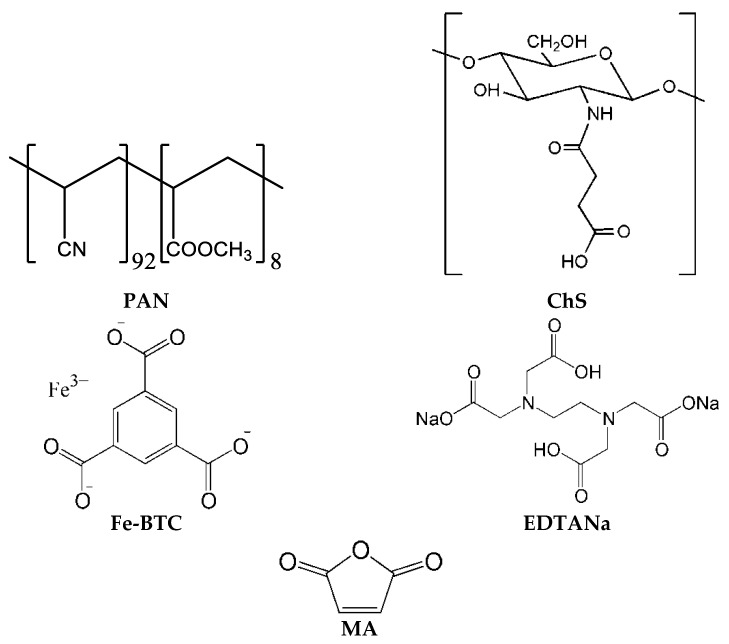
Structural formulas of used materials.

Ultrafiltration poly(acrylonitrile-co-methyl methacrylate) (PAN) membranes with molecular weight cut-off (MWCO) of 100 kDa and pure water flux 200–245 L m^−2^ h^−1^ (at transmembrane pressure 0.1 MPa) developed and produced by the Institute of Physical Organic Chemistry, National Academy of Sciences of Belarus (Minsk, Belarus) were used as a porous membrane support for preparation of thin film composite (TFC) and nanocomposite (TFN) membranes. Chitosan succinate (ChS, M_n_~30 10^3^ g mol^−1^, Bioprogress, Moscow, Russia) was selected for the formation of the selective layer of TFC and TFN membranes. Metal-organic frameworks (MOFs) based on iron 1,3,5-benzenetricarboxylate (Fe-BTC, Basolite^®®^ F300, Sigma Aldrich, St. Louis, MO, USA) were used as an additive to the ChS selective layer. The size of Fe-BTC agglomerates in the powder was studied by SEM and found to be 300–500 nm. Ethylene-diamine-tetra-acetic acid disodium salt (EDTANa, Sigma Aldrich, St. Louis, MO, USA) was used as a dispersing agent for Fe-BTC in aqueous ChS solutions. ChS was cross-linked using maleic anhydride (MA, Vekton, St. Petersburg, Russia) to prevent a selective layer swelling and dissolution during pervaporation. All materials were used without prior purification. Structural formulas of the used materials are presented in Figure 1.

### 2.2. Preparation of Thin Film Composite (TFC) and Thin Film Nanocomposite (TFN) Membranes

#### 2.2.1. Preparation of ChS-MA Solutions and ChS-MA-Fe-BTC Dispersions

Aqueous solutions containing 1.0 wt.% (for dynamic technique) and 2–3 wt.% (for physical adsorption technique) of ChS and 15 wt.% maleic anhydride (MA) with respect to ChS weight were prepared for the selective layer formation. The solutions were prepared by mixing a calculated amount of ChS in distilled water using a magnetic stirrer for 3 h at room temperature. Thereafter the MA was added and the solution was additionally mixed for 30 min. Then the solution was filtered using a glass Shott filter (pore size 60 µm).

The introduction of Fe-BTC (5–40 wt.% with respect to ChS weight) into the ChS aqueous solution was carried out in the following way. First, Fe-BTC was dispersed in distilled water using an ultrasound bath (Ultron, Olsztyn, Poland) for 30 min to obtain 2 wt.% aqueous dispersion. Separately, 5 wt.% EDTANa solution in distilled water was prepared by mixing for 30 min with a magnetic stirrer. A certain amount of Fe-BTC aqueous dispersion and EDTANa aqueous solution were mixed using a magnetic stirrer for 30 min and sonicated for 30 min. The weight ratio Fe-BTC:EDTANa was 1:2.5 correspondingly. After that, the Fe-BTC dispersion with EDTANa as a dispersing agent was added to ChS/MA aqueous solution and mixed for 10 min using a magnetic stirrer. Then, the resulting solution was sonicated for 30 min.

Above 20 wt.% Fe-BTC (with respect to ChS weight) for the physical adsorption technique (in 3 wt.% ChS aqueous solution) and 40 wt.% Fe-BTC for the dynamic technique (in 1 wt.% ChS aqueous solution), poor dispersion of the MOF particles in the membrane was observed and unreproducible transport characteristics were obtained.

#### 2.2.2. Formation of ChS Selective Layer via Dynamic Technique

The selective layers of TFC and TFN membranes were prepared via dead-end ultrafiltration of ChS/MA aqueous solution or ChS/Fe-BTC aqueous dispersion through PAN ultrafiltration membrane at transmembrane pressure of 3 bar. Two layers of ChS were deposited. The deposition time for the first layer was 10 min, and for the second layer 3 min. The first layer of composite membranes was kept for 2 h at room temperature, and then the second layer was applied. Thereafter, composite membranes were dried for 2 h at 110 °C in an oven, accompanied by crosslinking of ChS by MA. Membrane abbreviations and preparation conditions are listed in Table 1.

#### 2.2.3. Formation of ChS Selective Layer by Physical Adsorption

A sheet of PAN ultrafiltration membrane (15 × 20 cm in size) was washed from impregnating substances by soaking in distilled water for 12 h. Then it was tightly stretched over a metal ring so that the selective layer was located inside the ring. The stretched membrane was dried for 40 min. The ChS/MA aqueous solution or ChS/Fe-BTC aqueous dispersion was poured onto a selective layer of membranes, held for 10 s, and poured off. Next, the membrane was completely dried without removing it from the ring at room temperature for 2 h. Then, a second layer was applied in a similar way, but kept for 5 s. Thereafter, composite membranes were dried for 2 h at 110 °C in an oven, accompanied by crosslinking of ChS by MA. TFC and TFN membrane abbreviations and preparation conditions are presented in Table 1.

### 2.3. Membrane Characterization

#### 2.3.1. Fourier Transform Infrared Spectroscopy (FTIR)

The changes in composition of TFC and TFN membrane selective layer with addition of FeBTC were analyzed by FTIR spectrometer Nicolet Is50 (Thermofisher Scientific, Waltham, MA, USA) in the wavelength range 400–4000 cm^−1^ with accuracy 0.01 cm^−1^ at ambient temperature (25 °C).

#### 2.3.2. Scanning Electron Microscopy (SEM)

TFC and TFN membrane cross-section morphology was studied by Phenom Pro scanning electron microscope (Thermofisher Scientific, Waltham, MA, USA). Membranes were cleaved in liquid nitrogen followed by the coating of a gold layer using vacuum sputter coater DSR (Vaccoat, London, UK).

#### 2.3.3. Atomic Force Microscopy (AFM)

Topography of the surface of composite membranes was investigated using an NT MDT nTegra Maximus atomic force microscope (NT-MDT Spectrum Instruments, Zelenograd, Russia) with standard silicon cantilevers and with a stiffness of 15 N m^−1^.

#### 2.3.4. Contact Angle

Membrane water contact angles were determined by the sessile drop technique using a LK-1 goniometer (Otktrytaya Nauka, Krasnodar, Russia). Membrane sample was put on the flat surface, and a drop of water (1 µL) was placed on the surface. Measurements were taken in 5 s after the formation of a drop on the membrane surface. Measurements of 5 different samples of each membrane type were carried out and average value was calculated. Each membrane sample was measured three times. Measurement error did not exceed ±2°.

#### 2.3.5. Pervaporation Experiments

Membrane performance was studied by vacuum pervaporation for the separation of isopropanol/water mixtures with 12, 20 and 30 wt.% of water in the feed solution. The feed solution temperature was 25 °C. The pressure from permeate side was lower than 0.01 mmHg. The scheme of the pervaporation set-up is described in [47]. The feed and permeate compositions were analyzed by Chromatec Crystal 5000.2 gas chromatograph (Chromatec, Republic of Mari El, Yoshkar-Ola, Russia). Membrane permeation flux (J, g m^−2^ h^−1^) was calculated by Equation (1):

(1) $$J=\frac{m}{S\cdot t}$$

where m—the permeate weight, g; S—the effective membrane area, and m^2^; t—the time of pervaporation, h.

Moreover, the normalized flux (J_N_, g µm m^−2^ h^−1^) was counted by Equation (2):

(2) $$J_{N}=J\cdot l,$$

where l—selective layer thickness, µm. l was calculated from SEM micrographs of membrane selective layer.

Pervaporation separation index (PSI, kg m^−2^ h^−1^) was calculated by Equations (3) and (4):

(3) β=yAyBxAxB,

(4) $$\;\mathrm{PSI}=J\cdot \left(\beta -1\right),$$

where β—separation factor; J—permeation flux, kg m^−2^ h^−1^; *y_A_* and *y_B_*—water and alcohol concentration in the permeate, correspondingly; *x_A_* and *x_B_*—water and alcohol concentration in the feed solution, correspondingly.

Moreover, the thickness normalized pervaporation separation index (PSI_N_, kg µm m^−2^ h^−1^) was counted by Equation (5). PSI_N_ is a factor used to compare the pervaporation performance of various membranes in the same pervaporation process [54].

(5) $${\mathrm{PSI}}_{N}={\;J}_{N}\left(\beta -1\right),$$

where J_N_—normalized permeation flux, kg µm m^−2^ h^−1^.

## 3. Results and Discussions

In the present part, the transport properties and physico-chemical characteristics of novel thin film nanocomposite (TFN) chitosan succinate (ChS)/Fe-BTC membranes on poly-acrylonitrile (PAN) porous membrane-support obtained by two methods of selective layer formation (dynamic technique and physical adsorption) are presented.

### 3.1. Membrane Structure and Physicochemical Studies

#### 3.1.1. Studies of the Selective Layer Composition

**Figure 2:**
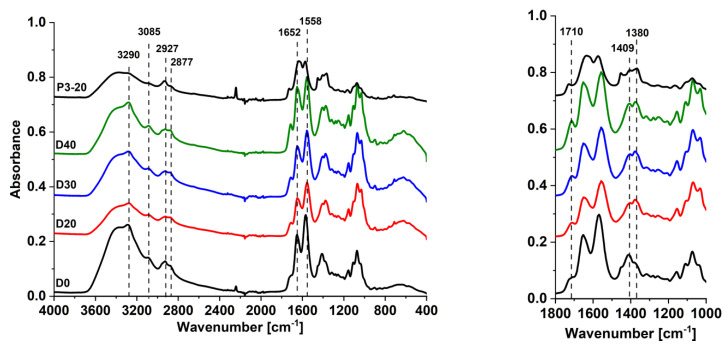
FTIR spectra of the surface of the selective layers of ChS/PAN (D0), TFN ChS-Fe-BTC/PAN membranes prepared by dynamic technique (D20, D30, D40) and physical adsorption (P3-20) technique.

FTIR spectra of selective layers of the reference TFC D0 membrane and TFN dynamic membranes D20, D30, D40, as well as P3-20 membrane obtained via physical adsorption, are presented in Figure 2.

Broad peak in the region of 3000–3600 cm^−1^ is attributed to the overlapping of stretching vibrations of hydroxyl groups (maximum at 3400 cm^−1^) and NH bonds of amide group (maximum at 3290 cm^−1^) in ChS (Figure 2). A shoulder at 3085 cm^−1^ is an overtone of the N–H bending band which is typical of primary amines. This can indicate that not all amine groups of chitosan were converted to amide groups in ChS. It was found that introduction of Fe-BTC in the ChS layer both for TFN membranes prepared by dynamic and physical adsorption technique yields a decrease in the intensity of the broad peak in the region of 3000–3600 cm^−1^ due to the overall decrease of ChS content in the selective layer (Figure 2).

Stretching vibrations NH-groups in the ChS are observed at 1558 cm^−1^ (Figure 1). Amide carboxyl stretching of ChS was observed at 1652 cm^−1^. Characteristic asymmetric fluctuations of the carboxylate groups of Fe-BTC (Figure 1) appeared at 1710 cm^−1^, and an increase in the MOFs concentration in the selective layer resulted in the increase in their intensity. Symmetrical fluctuations of the COO^−^-groups of Fe-BTC appeared at 1380 cm^−1^ when Fe-BTC was introduced to the selective layer [55,56]. Peaks at 1409 cm^−1^ are characteristic of the C=O bond, and peaks at 2877 and 2927 cm^−1^ are due to the symmetrical and asymmetrical stretching vibrations of CH_2_ groups.

So, the introduction of Fe-BTC in the ChS selective layer is confirmed by the increase of the intensity of peaks at 1710 cm^−1^ and appearance of the peak at 1380 cm^−1^.

#### 3.1.2. Scanning Electron Microscopy Studies

Morphology of the prepared composite membranes was investigated to describe transport properties in pervaporation since the change of surface and inner membrane structure significantly influence membrane performance. Moreover, it is known that membrane permeation flux depends on the selective layer thickness: increase in the selective layer thickness results in permeation flux decline due to the rise of the molecules pathway. The SEM microphotographs allow quantitatively determining the selective layer thickness.

**SEM of the dynamic composite membranes**

**Figure 3:**
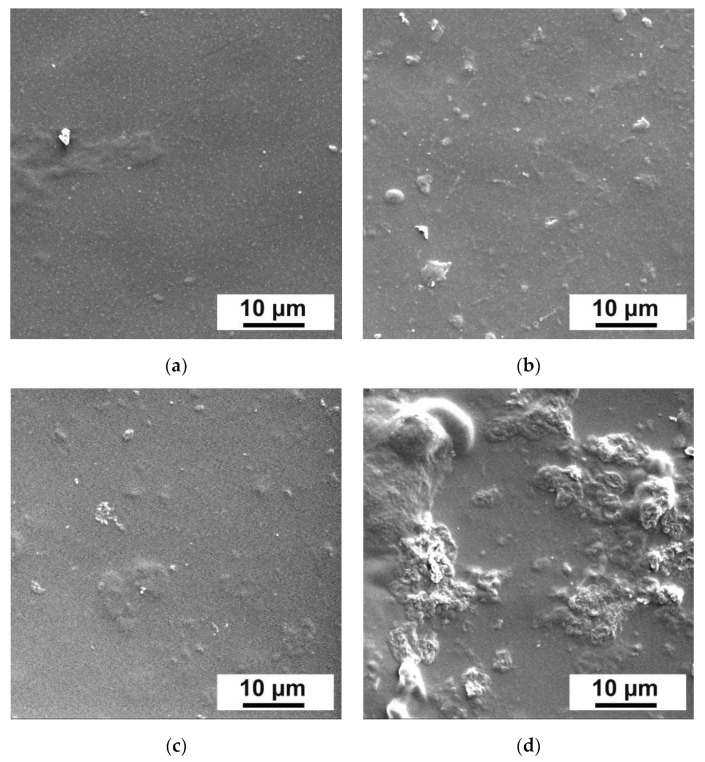
SEM microphotographs of the surface of selective layers of dynamic composite membranes: (**a**)—D0, (**b**)—D5, (**c**)—D20, (**d**)—D40.

Morphology of the surface of the membrane selective layers was determined by applying SEM (Figure 3).

It was shown that the morphology of the selective layer surface changed with addition of Fe-BTC into the selective layer. It was found that the Fe-BTC agglomerates formed on the selective layer surface and their size increased with increasing Fe-BTC concentration.

**Figure 4:**
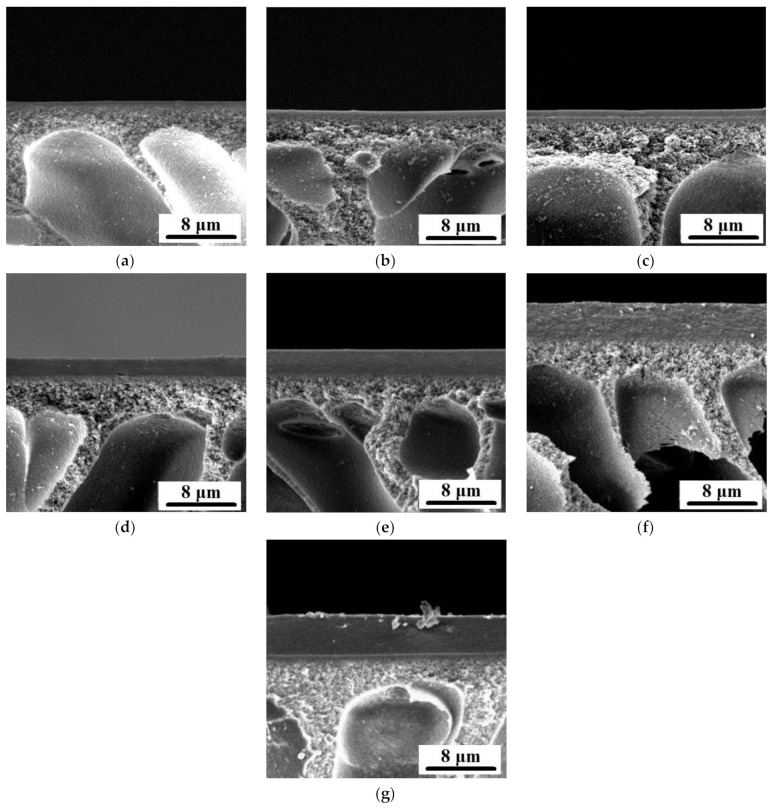
SEM microphotographs of the cross-sections of dynamic composite membranes: (**a**)—D0, (**b**)—D5, (**c**)—D10, (**d**)—D15, (**e**)—D20, (**f**)—D30, (**g**)—D40.

Microphotographs of dynamic composite membrane cross-sections are shown in Figure 4. Thickness of the selective layer of composite membrane was found from SEM microphotographs and presented in Table 2.

It was found that there was a tendency for the selective layer thickness of the modified membranes to increase with an increase in the concentration of Fe-BTC in the ChS selective layer (Figure 4, Table 2). Apparently, one of the reasons for such an effect is an increase in the free volume between the molecules that are formed in the selective layer with the addition of MOFs because of their structure. It was found that for D5 membranes thickness of the selective layer increased slightly (0.50 μm) compared to the selective layer thickness of the reference D0 membrane (0.44 μm). The selective layer thickness of D10 membrane was shown to be two times higher than that of the reference D0 membrane (Table 2).

Significant increase in the selective layer thickness was observed for membranes D15, D20, D30 and D40 (Table 2). It was found that the addition of 30–40 wt.% Fe-BTC/ChS weight into the ChS solution resulted in the increase in the thickness of the selective layer of thin film nanocomposite membrane by more than ten times compared to the reference ChS/PAN membrane D0 (Figure 4, Table 2). Increase in the selective layer thickness with addition of filler of dynamic TFN and TFC membranes was observed in previous studies [47,57].

Since TFN membranes were obtained via the dynamic mode, the concentration polarization mechanism determines the selective layer formation. The concentration of retained substances (ChS macromolecules and Fe-BTC particles) significantly increases in a thin boundary layer of solution near the membrane surface due to concentration polarization. When the concentration of retained substances and Fe-BTC particles reaches a certain value, the gel layer forms and precipitates on the membrane surface. This gel layer, after cross-linking with MA and drying, will form the membrane selective layer. So, dead-end ultrafiltration mode for selective layer formation was selected to enhance the concentration polarization phenomenon and decrease the time of gel layer formation on the membrane surface. Correlation between kinetics of gel layer formation, preparation conditions, and separation performance for TFC PVA/PAN membranes was revealed in our previous study [57]. It is worth noting that the gel layer formed on the membrane surface due to concentration polarization acts as a secondary barrier to flow through the membrane. Addition of Fe-BTC to the ChS aqueous solution yields hydrogen bonds formation between carboxylate groups of Fe-BTC and hydroxyl groups of ChS and donor-acceptor bonds formation between free orbitals of Fe atoms and lone pairs of electrons of oxygen atom in carboxyl and hydroxyl group in ChS. These bonds may provide additional cross-linking of the gel layer. This cross-linked gel layer hinders transport through the membrane, enhancing concentration polarization and leading to more macromolecules and particles accumulating near the membrane surface. Moreover, Fe-BTC particles are larger compared to ChS macromolecules and predominantly do not pass through the membrane accumulating in the gel layer. This increases the gel layer thickness. However, after some time of filtration a dynamic equilibrium between macromolecules and particles diffusing to the membrane surface and back to the bulk feed solution is established. Formation of hydrogen bonds and additional cross-linking retards the diffusion of ChS macromolecules back to the bulk solution and thicker gel layer is formed.

An increase in the selective layer thickness with an increase in the concentration of Fe-BTC in the ChS solution related also to the increase in the viscosity of the solution that was used for the selective layer formation. This leads to the formation of a thicker gel layer at the membrane surface due to concentration polarization during the formation of a selective layer in the dynamic technique.

**SEM of composite membranes prepared by physical adsorption**

**Figure 5:**
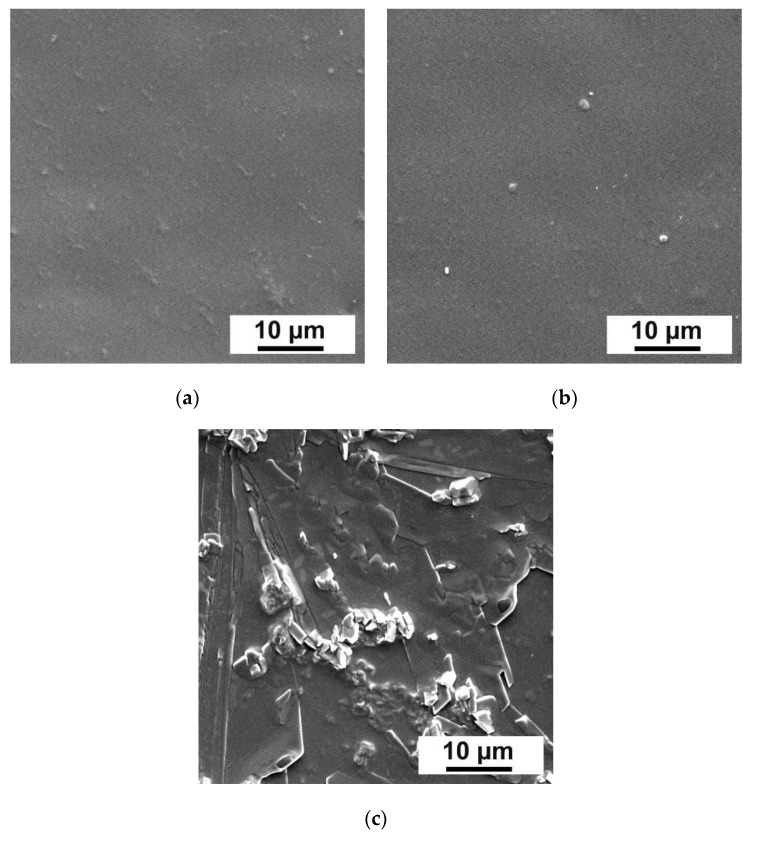
SEM microphotographs of selective layers of composite membranes ChS-Fe-BTC/PAN obtained by physical adsorption: (**a**) P3, (**b**) P3-5, (**c**) P3-20.

Morphology of membrane selective layers was determined by applying SEM (Figure 5).

It was found that introduction of MOFs into the selective layer led to the formation of nanoparticle agglomerates. Moreover, their size increased with the increase in the Fe-BTC concentration. Amount and size of agglomerates in the selective layer of P3-20 was revealed to be much higher compared to the same ChS/Fe-BTC ratio in the case of dynamic D20 TFN membrane (Figure 3). This is due to the lower dispersion degree of Fe-BTC in more concentrated ChS solution (3 wt.%) used for the preparation of membranes by physical adsorption technique compared to dynamic membranes. For preparation of dynamic membranes ChS solutions with lower concentration were used (1 wt.%) and thus Fe-BTC dispersion degree was higher.

**Figure 6:**
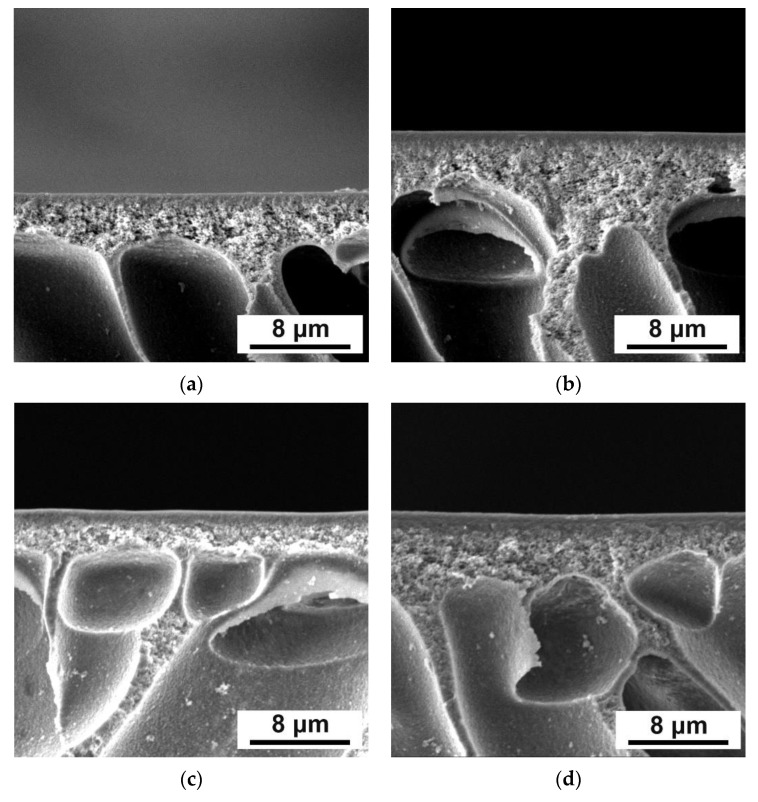
SEM microphotographs of the cross-sections of composite membranes ChS-Fe-BTC/PAN obtained by physical adsorption: (**a**) P3, (**b**)P3-5, (**c**) P3-10, (**d**) P3-20.

Morphology of membrane cross-sections was studied also for TFC ChS and TFN ChS/Fe-BTC membranes prepared by physical adsorption (Figure 6). It was found that selective layer thickness slightly increased even with addition of 20 wt.% Fe-BTC (Table 3).

Slight increase in the selective layer thickness should not significantly affect the nanocomposite membrane permeation flux. A significant difference in selective layer thickness of dynamic membranes and membranes prepared by physical adsorption is explained by the difference in mechanisms of the selective layer formation. The concentration polarization has a significant impact on the selective layer formation in the case of membranes obtained by dynamic technique. The boundary layer arises during filtration of solution, and upon reaching a certain concentration in it, the gel layer forms. This gel layer starts to grow during further filtration of modifying solution. In the case of physical adsorption, the selective layer is much thinner due to the adsorption of the particles on the membrane surface. Selective layer formation occurs due to the generation of hydrogen bonds, Van der Waals bonds or electrostatic interactions between the solution and membrane surface.

#### 3.1.3. Atomic Force Microscopy Studies of the Selective Layer Surface

**AFM of the dynamic composite membranes**

**Figure 7:**
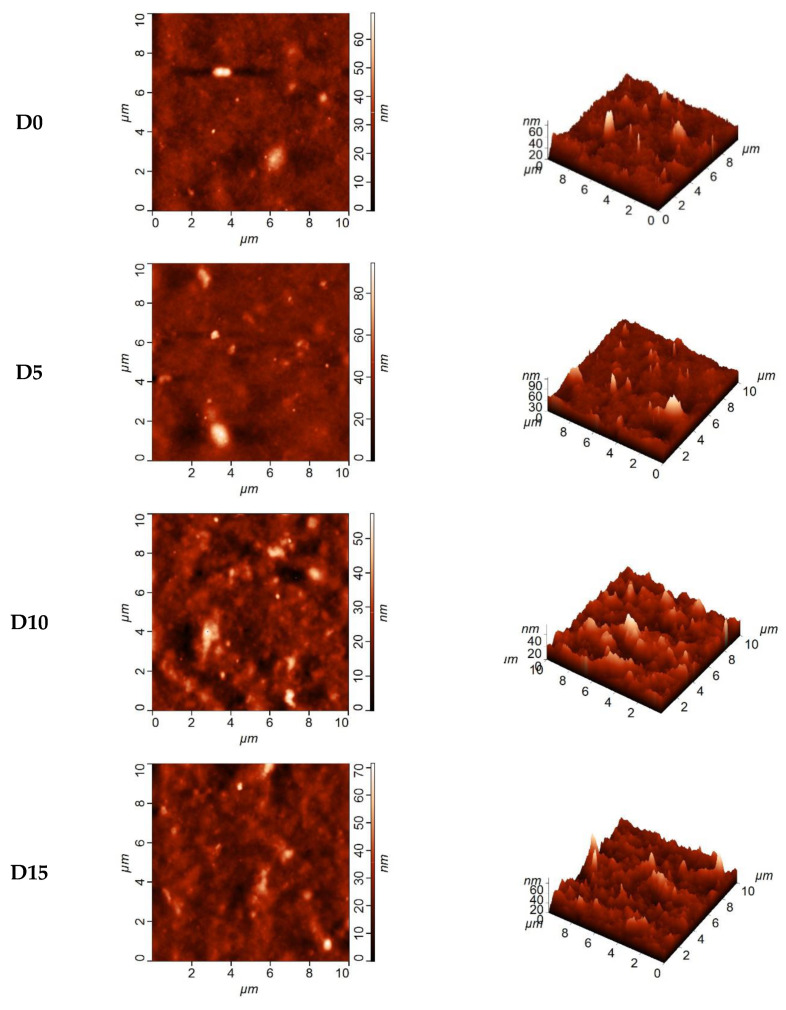

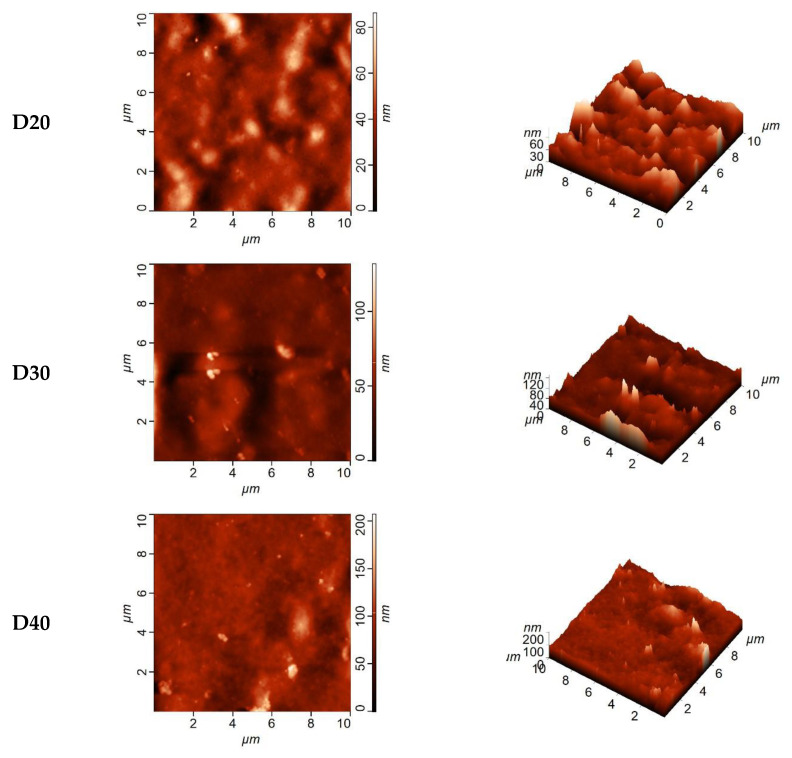
AFM images of the surface of the selective layer of TFC and TFN membranes prepared using dynamic technique.

Topology of the selective layer surface of dynamic composite membranes was investigated by AFM and presented in Figure 7.

It was shown that an increase in the concentration of Fe-BTC in the selective layer led to an increase in surface roughness (mean square roughness (R_q_) and average roughness (R_a_)) of thin film nanocomposite membranes compared to the initial membrane D0 (Figure 7, Table 4), while the surface of the reference D0 composite membrane was rather smooth.

According to Table 4, addition of Fe-BTC up to 15 wt.% into the modifying solution did not greatly affect the surface roughness. However, membranes D20, D30 and D40 are characterized by much higher surface roughness compared to the reference membrane D0. When the concentration of Fe-BTC increases, large globular domains of Fe-BTC can be observed on the surface of the membrane selective layer due to their agglomeration (Figure 7).

Globular domains could be responsible for the formation of the pattern on the membrane surface. Such selective layer structure provides higher permeation flux of the prepared composite and nanocomposite membranes in pervaporation.

**AFM of composite membranes prepared by physical adsorption**

**Figure 8:**
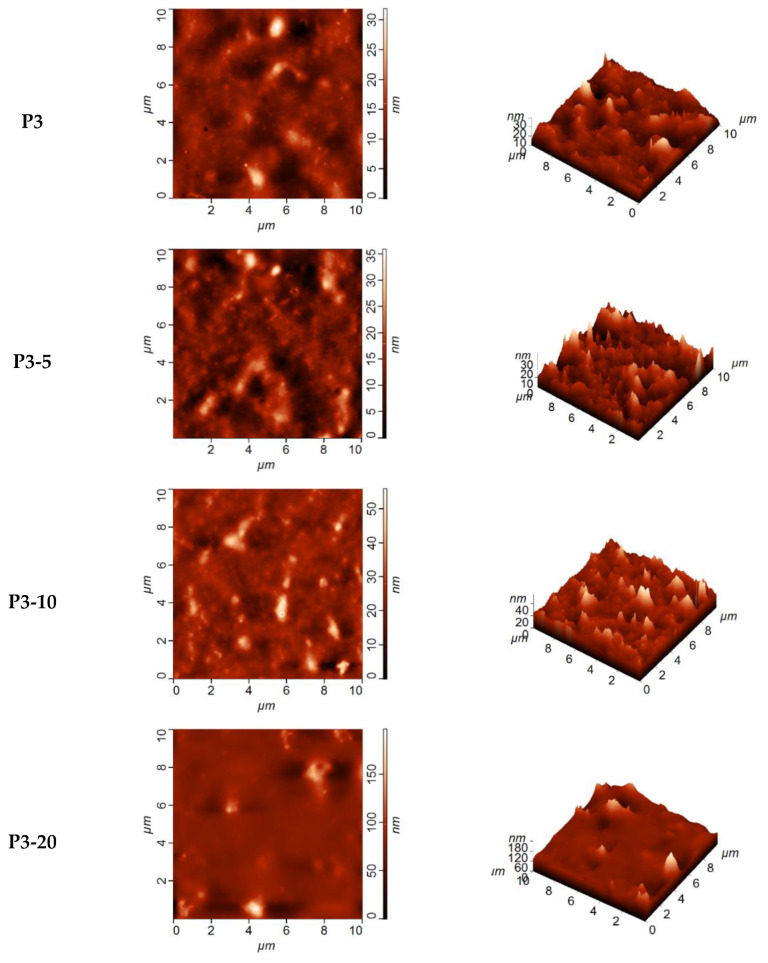
AFM images of the surface of the selective layer of TFC and TFN membranes prepared by physical adsorption.

Topology investigated by AFM of the selective layer surface of TFN membranes prepared by physical adsorption is presented in Figure 8.

Figure 8 shows that there were aggregates that were formed on the selective layer surface when introducing Fe-BTC. Based on AFM images mean square roughness (R_q_) and average roughness (R_a_)) were calculated (Table 5).

It was shown that surface roughness increased for membranes prepared by physical adsorption, similar to the dynamic membranes. It should be noted that the average roughness of membranes obtained by physical adsorption was lower compared to the dynamic composite membranes. This is due to the peculiarities of the mechanism of gel layer formation during preparation of dynamic membranes. After a certain time of filtration a dynamic equilibrium between macromolecules and particles diffusing to the membrane surface and back to the bulk feed solution is established. Due to this, a concentration gradient in the boundary layer is formed which leads to the formation of the selective layer with looser and less ordered structure compared to the selective layer of membranes obtained via physical adsorption.

However, if we compare AFM images of both types of TFN membranes with similar Fe-BTC concentration in the selective layer, it can be noted that Fe-BTC particles form larger agglomerates which are less uniformly dispersed for membranes prepared via physical adsorption compared to the dynamic membranes (Figure 7 and Figure 8). Lower dispersion degree of Fe-BTC particles compared to the similar concentration of Fe-BTC for dynamic membranes is attributed to the higher concentration of ChS aqueous solution (and thus higher viscosity) used for the selective layer formation by physical adsorption (3 wt.%) compared to the dynamic membranes (1 wt.%).

#### 3.1.4. Contact Angle

**Figure 9:**
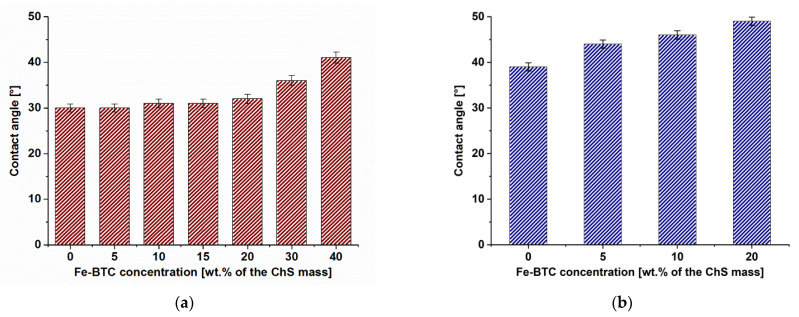
Dependence of water contact angle of the selective layer surface of ChS-Fe-BTC/PAN TFN membranes on Fe-BTC concentration: (**a**)—dynamic membranes, (**b**)—membranes prepared by physical adsorption.

Another technique that helps to evaluate the change of the membrane surface after its modification is evaluation of water contact angles. Dependence of contact angle of the selective layer surface of ChS-Fe-BTC/PAN TFN membranes on Fe-BTC concentration is presented in Figure 9.

The contact angle was shown to slightly increase with rise of MOFs concentration in the selective layer both for dynamic membranes and membranes prepared by physical adsorption.

For dynamic membranes it was found that contact angle of the selective layer surface of the reference D0 membrane was 30 ± 2°. It was revealed that introduction of 5 wt.% Fe-BTC in ChS solution does not change the hydrophilic-hydrophobic properties of membrane selective layer. It was shown that the contact angle increases slightly with addition of 10–20 wt.% Fe-BTC into the selective layer. The highest water contact angle of 40 ± 2 is observed for D40 membrane.

The contact angles of membranes prepared by physical adsorption were revealed to increase from 39° for P3 up to 49° for P3-20 TFN membranes. An increase in the contact angle indicates the hydrophobization of the selective layer surface of thin nanocomposite membranes, due to the presence of Fe-BTC hydrophobic units in the selective layer. Another reason for the increase of contact angle is the increase in the degree of surface roughness (Table 4 and Table 5) due to the formation of agglomerates of Fe-BTC on the surface of membrane selective layer (Figure 7 and Figure 8) which was revealed for both types of TFN membranes. However, it is worth noting that, in spite of the increase of contact angle from 30 to 40°, the TFN ChS-Fe-BTC/PAN membranes are considered to be moderately hydrophilic.

Higher water contact angle for the reference TFC ChS/PAN membrane prepared via physical adsorption compared to the reference dynamic membrane was a result of different mechanisms of selective layer formation. The formation of the selective layer of dynamic membranes occurs due to the filtration of the ChS aqueous solution through the porous membrane support at transmembrane pressure of 3 bar. It is likely that elevated pressure may decrease the cross-linking degree of ChS macromolecular chains which is likely to increase the hydrophilicity of the membrane as was observed in the previous study [57].

Higher water contact angles of the selective layer of ChS-Fe-BTC/PAN TFN membranes prepared via physical adsorption compared to the dynamic membranes is also attributed to the different mechanism of selective layer formation. In the case of dynamic technique, larger Fe-BTC particles were predominantly concentrated on the boundary layer near the membrane surface because they are almost completely rejected by the membrane and their diffusion back to the bulk solution is restricted due to the large size. Thus, more hydrophilic ChS macromolecules appeared on the surface of the selective layer of TFN dynamic membranes providing lower water contact angle.

Higher contact angle of the surface of TFN membranes prepared by physical adsorption was due to the lower dispersion degree of Fe-BTC particles compared to the similar concentration of Fe-BTC for dynamic membranes which was discussed in Section 3.1.3. Lower dispersion degree is attributed to the higher concentration of ChS aqueous solution (and thus higher viscosity) used for the selective layer formation by physical adsorption (3 wt.%) compared to the dynamic membranes (1 wt.%).

### 3.2. Membrane Pervaporation Performance

#### 3.2.1. Performance of Thin Film Composite Membranes Prepared via Dynamic Technique

**Figure 10:**
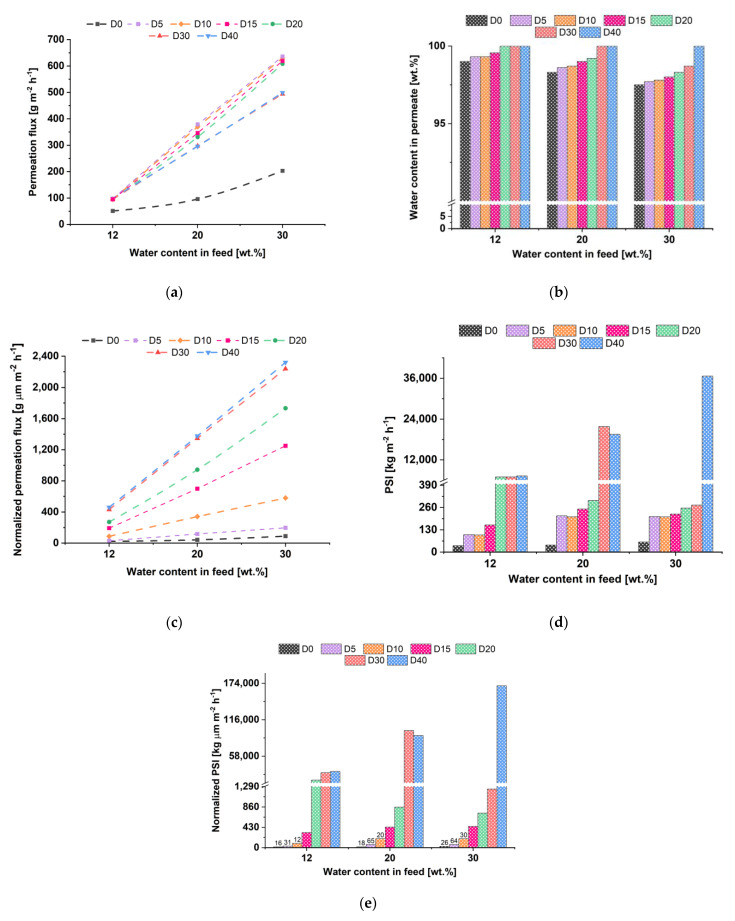
The dependence of permeation flux (**a**), water content in permeate (**b**), PSI (**c**), normalized permeation flux (**d**) and normalized PSI (**e**) of dynamic TFC membranes with different Fe-BTC concentration in the ChS selective layer on the water content in isopropanol/water feed solutions.

The transport properties of membranes were studied in the pervaporation of a water/isopropyl alcohol mixture (12–30 wt.% water). The choice of this system was due to the importance of purified iPrOH as an industrial solvent actively applied instead of ethanol in various fields [11]. The separation of water/iPrOH mixture by traditional methods (rectification and distillation) is complicated due to the formation of an azeotrope with a water content of 12 wt.% [12]. Traditional separation methods are energetically unfavorable and require the addition of toxic intermediates to form stronger azeotropic mixtures with water, contributing to an additional alcohol purification stage. However, this separation problem may be easily solved by an environmentally friendly separation method—pervaporation using the chitosan succinate-based membranes with high hydrophilicity, which promotes the preferential penetration of water molecules (high selective water removal), high efficiency and productivity of the process. The dependence of the permeation flux, water content in the permeate, pervaporation separation index and normalized permeation flux on the water content in the feed are presented in Figure 10.

Studies of TFC and TFN membrane performance in pervaporation separation of 88 wt.% isopropanol/12 wt.% water mixture reveal that permeation flux of ChS-Fe-BTC/PAN TFN membranes was two times higher than that of the reference ChS/PAN (D0) membrane (Figure 10a). It was shown that the water content in the permeate for membrane D0 was 99 wt.% and for membranes D20, D30 and D40—99.99 wt.% (Figure 10b). This indicates a high selectivity toward water of both the ChS/PAN and ChS-Fe-BTC/PAN membranes.

Increase of water content in the feed solution was found to yield an increase in the permeation flux of both reference TFC (D0) and ChS-Fe-BTC/PAN TFN membranes. This is due to the swelling of the selective layer in the feed solution during pervaporation as well as higher degree of water sorption on the surface of the membrane selective layer due to higher concentration of water molecules in the mixture. It was found that, with an increase in Fe-BTC concentration in the ChS solution, permeation flux increases non-monotonically. It was shown that D5 membrane is characterized by the highest permeation flux at 20 and 30 wt.% water in the feed (Figure 10a). The substantial rise in permeation flux when 5 wt.% of Fe-BTC is added to the solution compared to the reference membrane is attributed to the formation of a less dense selective layer (less dense packing of polymer chains) due to Fe-BTC agglomerates. The water content in permeate monotonically increases with an increase in Fe-BTC concentration in the selective layer when dehydrating isopropanol with 20 and 30 wt.% water (Figure 10b). It was revealed that increasing of the water content in the feed solution results in the decreasing of water content in permeate both for reference ChS/PAN thin film composite membrane and for ChS-Fe-BTC/PAN thin film nanocomposite membranes. However, the D30 and D40 membranes were found to have higher selectivity in pervaporation of more diluted isopropanol aqueous solutions (20 wt.% and 30 wt.% of water) compared to the reference D0 and other TFN membranes. Nevertheless, D40 membrane had a high selectivity in the pervaporation of the isopropanol/water mixtures with the water content in feed up to 30 wt.% and characterized by constant value of water content in permeate (99.99 wt.%) (Figure 10b).

It was revealed that the normalized flux of dynamic TFN membranes rose with increase in the Fe-BTC concentration (Figure 10c). Normalized flux was counted taking into account the thickness of the selective layer.

It was found that membrane modification by addition of Fe-BTC in the selective layer leads to the significant increase in PSI and normalized PSI for all studied TFN membranes due to the simultaneous increasing in permeation flux and selectivity (Figure 10d,e). This is caused by the amorpho-crystalline structure of Fe-BTC and the presence of the size-defined regions. On the one hand, it increases the free volume of the selective layer due to decrease of polymer chains packing density and, therefore, permeation flux increases. Moreover, presence of Fe-BTC in the selective layer structure yields the rise in the difference in the diffusion rates of the molecules with various sizes through the selective layer due to the increase in the length of the diffusion path and the presence of regions with size-defined parameters. It is also likely that there are additional crosslinking and stabilization of the ChS structure with the introduction of Fe-BTC in the selective layer due to the formation of donor-acceptor bonds of iron atoms of Fe-BTC and hydroxyl and/or carboxyl groups of ChS. Additional crosslinking may be also due to the formation of hydrogen bonds between the carbonyl groups of trimesoyl chloride in the structure of Fe-BTC and hydroxyl groups of ChS. It was found that membrane D40 demonstrates the highest PSI and normalized PSI values at 12 wt.% and 30 wt.% water in the feed solution, but D30 is the most effective in pervaporation of the mixture 20 wt.% isopropanol/80 wt.% water (Figure 10d). It was found that normalized PSI (Figure 10e) was found to correlate with the PSI values (Figure 10d) and showed that TFN dynamic membranes were more effective than TFC D0 membrane. Moreover, the nanocomposite D40 membrane possessed the best normalized PSI value.

#### 3.2.2. Performance of Membranes Prepared by Physical Adsorption

**Performance of thin film composite membranes**

**Figure 11:**
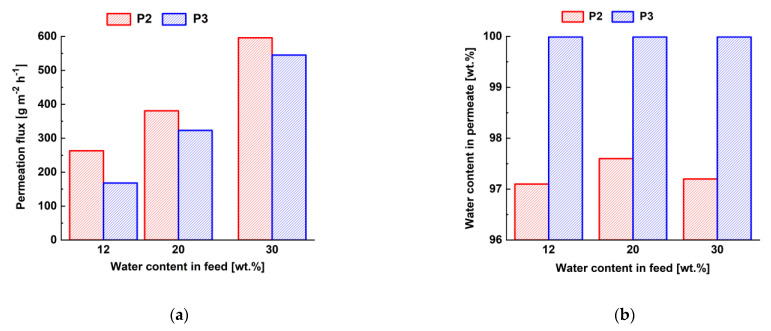
Dependence of permeation flux (**a**) and water content in permeate (**b**) on the water content in feed mixture during the pervaporation of isopropanol/water mixture (12–30 wt.% water) for TFC ChS/PAN membrane prepared by physical adsorption.

To select the optimal concentration of ChS aqueous solution, the pervaporation performance of the composite membranes prepared by the physical adsorption using two concentrations of chitosan succinate was studied. Figure 11 shows the dependence of permeation flux and the water content in the permeate on the water content in feed mixture in pervaporation of isopropanol/water mixture with different amounts of water (12–30 wt.%).

It was found that membrane permeation flux slightly decreased along with increase in the ChS concentration in the solution (Figure 11a) in dehydration of isopropanol/water. Moreover, higher water concentration in feed solution resulted in the significant increase in membrane permeation flux (Figure 11a) due to the selective layer swelling. It was shown that selectivity of P3 composite membrane is higher than that for P2 composite membranes (Figure 11b). Moreover, the selectivity of P3 membrane was found to be constant with increase in water concentration in feed. Thus, 3 wt.% ChS aqueous solution was selected for further investigations due to the high permeation flux and selectivity of the corresponding composite membrane.

**Performance of TFN membranes prepared by physical adsorption**

**Figure 12:**
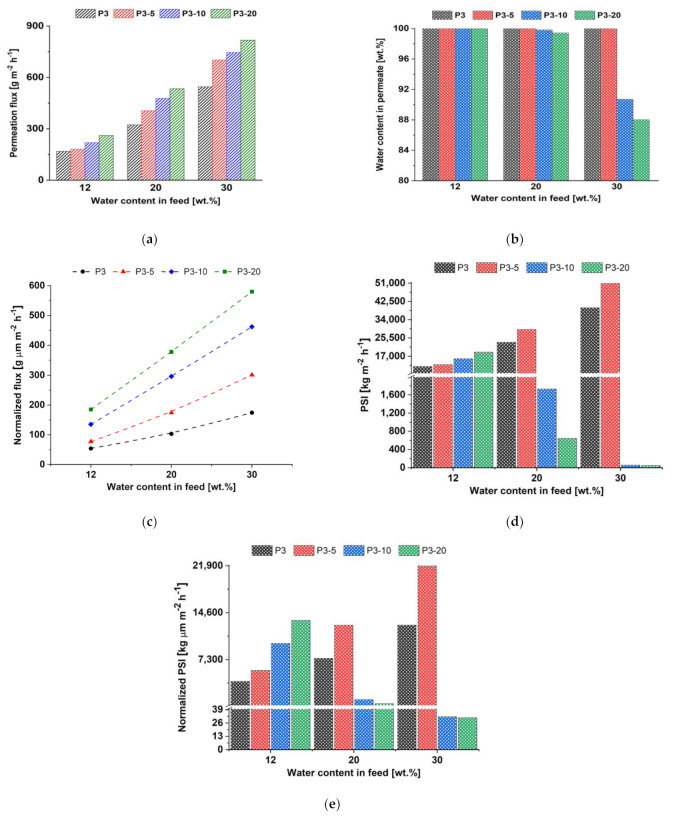
Dependence of permeation flux (**a**), water content in permeate (**b**), normalized permeation flux (**c**), PSI (**d**) and normalized PSI (**e**) on the water content in feed solution in pervaporation of isopropanol/water mixture for ChS-Fe-BTC/PAN membranes prepared by physical adsorption with different content of Fe-BTC in the selective layer.

In order to investigate the effect of Fe-BTC concentration on the TFN membrane performance, 5, 10 and 20 wt.% of Fe-BTC was introduced in to the selective layer. Performance of the developed TFN membranes was studied in pervaporation of isopropanol/water feed mixture with 12, 20 and 30 wt.% water. The dependences of permeation flux, water content in the permeate, normalized permeation flux, PSI and normalized PSI on the water content in feed solution are shown in Figure 12.

It was found that the introduction of Fe-BTC into the ChS selective layer led to an increase in permeation flux from 168–545 g m^−2^ h^−1^ for P3 membrane to 260–817 g m^−2^ h^−1^ for P3-20 TFN membrane in pervaporation of isopropanol/water mixture (12–30 wt.% water). The reason for the permeation flux increase was the same as that for dynamic TFN membranes. An increase in permeation flux was noted along with an increase in the water content in feed due to the swelling of the selective layer in more dilute solutions (Figure 12a). However, an increase in permeation flux did not affect the selectivity of TFN membranes in dehydration of isopropanol with 12 wt.% water (Figure 12b). It was shown that further increase in the water content in feed solution resulted in loss of membrane selectivity, with the exception of P3-5 TFN membrane. TFN membranes P3-10 and P3-20 demonstrated lower selectivity (92 wt.% water in permeate) in pervaporation of isopropanol/water mixture with 30 wt.% water. This means that membranes prepared by physical adsorption are characterized by a less cross-linked layer that led to the higher swelling. Moreover, due to a lower degree of Fe-BTC dispersion for membranes prepared by physical adsorption compared to the dynamic membranes, and the presence of large Fe-BTC agglomerates, imperfections of the selective layer may arise at the ChS-Fe-BTC interface. Normalized flux was shown to increase with addition of Fe-BTC into the ChS selective layer (Figure 12c). PSI values (Figure 12d) confirmed that P3-5 TFN membrane was more effective than P3, P3-10 and P3-20 membranes in pervaporation separation of investigated isopropanol/water mixtures. Figure 10e showed that thickness of normalized PSI was lower compared to PSI (Figure 12c). However, it correlated with PSI, and it was confirmed that P3-5 was more stable in pervaporation of isopropanol with 20 and 30 wt.% water, although P3-20 TFN membrane possessed the highest thickness normalized PSI in pervaporation 88 wt.% isopropanol/12 wt.% water feed mixture. Based on the data presented in Figure 12, the optimal concentration of Fe-BTC in the ChS for the membranes prepared by physical adsorption was found to be 5 wt.%.

### 3.3. Comparison of D40 and P3-5 TFN Membranes

Thus, based on the above-described results it can be concluded that TFN membranes D40 and P3-5 were found to be the most effective in isopropanol/water pervaporation (Figure 10 and Figure 12). Comparison of TFN membrane characteristics is presented in Table 6.

It was found that both types of membranes are effective in the dehydration of isopropanol. Higher permeation fluxes were obtained for membranes prepared by physical adsorption due to the smaller thickness of the selective layer. However, the structure of the selective layer is different for the selective layers prepared by dynamic and physical adsorption techniques. According to the normalized flux and normalized PSI, it can be concluded that a layer with higher free volume and less dense packing of polymer chains is formed in the case of dynamic membranes without deterioration in membrane selectivity (Figure 10c,e and Figure 12c,e and Table 6). Moreover, increase of Fe-BTC concentration in the selective layer of dynamic membranes leads to the formation of more stable membranes which are less prone to swelling with the increase of water content in the feed compared to the TFN membranes prepared by physical adsorption (Figure 9b and Figure 11b). These differences of selective layer structure and pervaporation performance are due to the different mechanisms of selective layer formation which are based on different physical phenomena—formation of gel layer due to concentration polarization and physical adsorption. The advantage of the dynamic mode of selective layer formation is the possibility of obtaining TFC and TFN membranes via modification of commercial ultrafiltration modules, which is technologically more feasible and prevents the damage of the selective layer during technological operation. Dynamic membranes are more stable in feed mixtures with higher water content compared to the membranes obtained via physical adsorption. However, it is necessary to decrease the thickness of the selective layer of dynamic membranes to increase the permeation flux.

**Figure 13:**
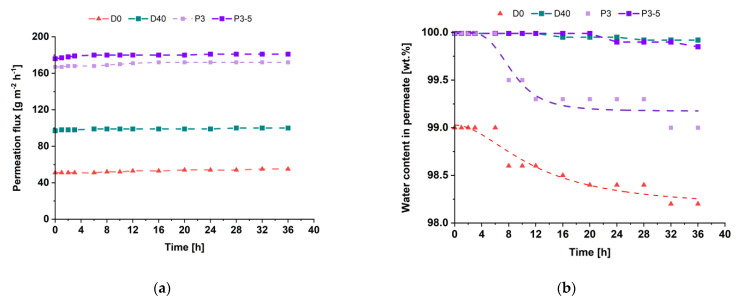
The long-term performance of the D0, D40, P3 and P3-5 membranes in pervaporation of 88 wt.% isopropanol/12 wt.% water mixture at 25 °C: (**a**)—permeation flux, (**b**)—water content in permeate.

To evaluate from the perspective of industrial application, the stability of the D0, D40, P3 and P3-5 membranes was investigated during 36 h of pervaporation (Figure 13).

It was shown that both D40 and P3-5 TFN membranes were stable in the long-term experiment during pervaporation of 88 wt.% isopropanol/12 wt.% water mixture at 25 °C. It confirmed the formation of a dense defect-free selective layer when Fe-BTC is added. Moreover, TFN membranes were revealed to be more stable to swelling and thus demonstrated constant water content in permeate compared to the reference membranes, for which a slight decrease of water content in permeate was observed.

### 3.4. Comparison of the Performance of the Developed TFN Membranes with Chitosan-Based Membranes

Transport properties of D40 and P3-5 TFN membranes were compared with the literature data on chitosan-based membranes applied for the dehydration of isopropanol in pervaporation process at comparable conditions to the present study. The comparison is presented in Table 7.

The data presented in Table 7 demonstrate that the developed D40 and P3-5 TFN membranes exhibit advanced transport properties, either a higher permeation flux or separation factor (73,326), compared to other chitosan-based membranes used for pervaporation dehydration of isopropanol. It is also worth noting that the developed P3-5 TFN membrane has a permeation flux ~6.5 times higher with the same level of separation factor (73,326) than that for the commercial membrane PERVAP^TM^ 1201 (Sulzer, Chemtech, Switzerland) [9] in pervaporation of a azeotrope water/isopropanol (12/88 wt.%) mixture.

## 4. Conclusions

Novel TFN membranes for pervaporation based on chitosan succinate modified with Fe-BTC on a porous PAN membrane-support were developed by two different techniques: dynamic technique and physical adsorption. It was found that an increase in the concentration of Fe-BTC in the ChS caused (1) an increase of the selective layer thickness due to the rise in the viscosity of solutions (confirmed by SEM); (2) an increase in surface roughness of TFN membranes compared to the reference membranes which was the result of formation of large globular aggregates of Fe-BTC on the selective layer membrane surface (confirmed by AFM); (3) increase in contact angle due to the presence of more hydrophobic Fe-BTC units. It was revealed that membrane modification by addition of Fe-BTC in the selective layer led to a rise in TFN membrane permeation flux, as well as normalized flux, selectivity and PSI. Optimal concentrations of Fe-BTC for effective pervaporation TFN membranes were revealed to be 40 and 5 wt.% for dynamic technique and physical adsorption membrane preparation techniques, correspondingly. On the base of the described results, it can be concluded that the developed supported pervaporation membranes can be used for the separation of industrially significant liquid mixtures by the pervaporation method (dehydration of alcohols and organic solvents), in oil refining, chemical, pharmaceutical and food industries.

## Figures and Tables

**Table 1:** TFC and TFN membrane abbreviations and preparation conditions.

Abbreviation	ChS Concentration in Aqueous Solution [wt.%]	Fe-BTC Concentration in ChS Solution [wt.%, Ratio to ChS]	Method of the Selective Layer Formation
D0	1.0	0	dynamic technique
D5		5	
D10		10	
D15		15	
D20		20	
D30		30	
D40		40	
P2	2	0	physical adsorption
P3	3	0	
P3-5		5	
P3-10		10	
P3-20		20	

**Table 2:** Thickness of the selective layer of dynamic composite membranes according to SEM microphotographs.

Membrane Abbreviation	Selective Layer Thickness [μm]
D0	0.44
D5	0.50
D10	0.92
D15	2.02
D20	2.85
D30	4.53
D40	4.65

**Table 3:** Thickness of the selective layer of composite membranes obtained by physical adsorption according to SEM microphotographs.

Membrane Abbreviation	Selective Layer Thickness [μm]
P3	0.32
P3-5	0.43
P3-10	0.62
P3-20	0.71

**Table 4:** Surface roughness parameters of the selective layer surface of TFC and TFN membranes prepared using dynamic technique.

Membrane Abbreviation	Roughness Parameters	
	R_a_ [nm]	R_q_ [nm]
D0	3.44	5.07
D5	4.46	5.97
D10	4.61	6.07
D15	4.63	6.39
D20	8.25	10.87
D30	8.95	11.74
D40	10.39	14.88

**Table 5:** Surface roughness parameters of the selective layer surface of TFC and TFN membranes prepared using physical adsorption.

Membrane Abbreviation	Roughness Parameters	
	R_a_ [nm]	R_q_ [nm]
P3	2.31	3.20
P3-5	3.40	4.48
P3-10	3.58	5.13
P3-20	7.73	13.16

**Table 6:** Comparison of D40 and P3-5 TFN membrane characteristics.

	Membrane Abbreviation	D40	P3-5
Parameter			
Selective Layer Thickness [µm]		4.65	0.43
Roughness parameters	R_a_ [nm]	10.39	3.40
	R_q_ [nm]	14.88	4.48
Contact angle [°]		41	44
Permeation flux (g m^−2^ h^−1^)	88% isopropanol/12% water	99	180
	80% isopropanol/20% water	296	405
	70% isopropanol/30% water	499	701
Normalized flux (g µm m^−2^ h^−1^)	88% isopropanol/12% water	460	77
	80% isopropanol/20% water	1376	174
	70% isopropanol/30% water	2320	301
Water content in permeate (wt.%)	88% isopropanol/12% water	99.99	99.99
	80% isopropanol/20% water	99.99	99.99
	70% isopropanol/30% water	99.99	99.99
PSI (kg m^−2^ h^−1^)	88% isopropanol/12% water	7,259	13,050
	80% isopropanol/20% water	19,502	29,362
	70% isopropanol/30% water	36,589	50,822
Normalized PSI (kg µm m^−2^ h^−1^)	88% isopropanol/12% water	33,754	5,612
	80% isopropanol/20% water	90,684	12,626
	70% isopropanol/30% water	170,139	21,853

**Table 7:** Comparison of the transport properties of D40 and P3-5 TFN membranes for isopropanol dehydration by pervaporation.

Membranes	Thickness (µm)	Water Content in Feed (wt.%)	Temperature (°C)	Permeation Flux (g m^−2^ h^−1^)	Separation Factor (β)	References
D40	4.65	12	25	99	73,326	This study
P3-5	0.43	12	25	180	73,326	This study
PERVAP^TM^ 1201	-	12	22	28	73,326	[9]
Chitosan/polyvinyl alcohol (20 wt.%)	35-40	15	30	130	1625	[19]
Chitosan/gelatin (15 wt.%)	-	10	30	42	6330	[58]
Polyelectrolyte complex membranes from chitosan and polystyrene sulfonic acid-co-maleic acid (9 wt.%)	40	~12	30	29	2898	[59]
Supported chitosan membrane	~0.6–0.7	12	28	130	~200	[26]
Chitosan (1 wt.%)–Graphene oxide (0.1 wt.%)/trimesoyl chloride/3 cycles	0.432	10	60	4391	1491	[60]
Chitosan (1 wt.%)–Graphene oxide (0.2 wt.%)/trimesoyl chloride/3 cycles	0.682	10	60	2835	2991	[60]
Chitosan/hydroxy-ethyl-cellulose (CS/HEC)	30–35	10	60	175	26,091	[61]
Chitosan/Cellulose Acetate composite hollow fiber membranes	-	10	25	166	~809	[62]
Chitosan/NaY zeolite (30 wt.%)	40	5	30	115	2620	[63]
Chitosan cross-linked with sulfo-succinic acid	20	20	40	105	∞	[64]
Chitosan cross-linked with toluene-2,4-diisocyanate	50	8.4	30	79	472	[65]
Chitosan/NH_4_Y zeolite (0.2 wt.%)	30	10	30	39	~38	[66]
Chitosan/polyvinyl alcohol (75/25)	18–25	10	60	644	∞	[67]
Supported chitosan membrane	~1.26	10	25	409	1490	[68]
Chitosan/blocked diisocyanate (40 wt.%)	40	10	30	34	2423	[69]
Chitosan/hydroxy-propyl cellulose (40 wt.%)	50	12.5	30	263	320	[70]
Chitosan (1.5 wt.%)/poly-sulfone composite hollow fiber membranes	~0.7	30	25	128	78	[71]
Chitosan/Na^+^-MMT clay (10 wt.%)	40	10	30	14.23	14,992	[72]
Polyelectrolyte complex membranes from chitosan and phospho-tungstic acid (0.045 M)	40	10	30	1170	7490	[73]
Chitosan-g-polyaniline	40	10	30	19	502	[74]

## Data Availability

The data presented in this study are available on request from the corresponding author. The data are not publicly available due to being a part of ongoing research.

## References

[1] Abdin A.Y., Yeboah P., Jacob C. (2020). Chemical Impurities: An Epistemological Riddle with Serious Side Effects. IJERPH Int. J. Environ. Res. Public Health.

[2] Atlaskin A.A., Trubyanov M.M., Yanbikov N.R., Bukovsky M.V., Drozdov P.N., Vorotyntsev V.M., Vorotyntsev I.V. (2018). Total Reflux Operating Mode of Apparatuses of a Membrane Column Type during High Purification of Gases to Remove a Highly Permeable Impurity. Pet. Chem..

[3] Atlaskin A.A., Trubyanov M.M., Yanbikov N.R., Vorotyntsev A.V., Drozdov P.N., Vorotyntsev V.M., Vorotyntsev I.V. (2019). Comprehensive Experimental Study of Membrane Cascades Type of “Continuous Membrane Column” for Gases High-Purification. J. Membr. Sci..

[4] Davletbaeva I., Zaripov I., Mazilnikov A., Davletbaev R., Sharifullin R., Atlaskin A., Sazanova T., Vorotyntsev I. (2019). Synthesis and Study of Gas Transport Properties of Polymers Based on Macroinitiators and 2,4-Toluene Diisocyanate. Membranes.

[5] Besha A.T., Tsehaye M.T., Tiruye G.A., Gebreyohannes A.Y., Awoke A., Tufa R.A. (2020). Deployable Membrane-Based Energy Technologies: The Ethiopian Prospect. Sustainability.

[6] Gui S., Mai Z., Fu J., Wei Y., Wan J. (2020). Transport Models of Ammonium Nitrogen in Wastewater from Rare Earth Smelteries by Reverse Osmosis Membranes. Sustainability.

[7] Yushkin A.A., Golubev G.S., Podtynnikov I.A., Borisov I.L., Volkov V.V., Volkov A.V. (2020). Separation of Mixtures of Polar and Nonpolar Organic Liquids by Pervaporation and Nanofiltration (Review). Pet. Chem..

[8] Apel P.Y., Bobreshova O.V., Volkov A.V., Volkov V.V., Nikonenko V.V., Stenina I.A., Filippov A.N., Yampolskii Y.P., Yaroslavtsev A.B. (2019). Prospects of Membrane Science Development. Membr. Membr. Technol..

[9] Dmitrenko M., Liamin V., Kuzminova A., Mazur A., Lahderanta E., Ermakov S., Penkova A. (2020). Novel Mixed Matrix Sodium Alginate–Fullerenol Membranes: Development, Characterization, and Study in Pervaporation Dehydration of Isopropanol. Polymers.

[10] Jyothi M.S., Reddy K.R., Soontarapa K., Naveen S., Raghu A.V., Kulkarni R.V., Suhas D.P., Shetti N.P., Nadagouda M.N., Aminabhavi T.M. (2019). Membranes for Dehydration of Alcohols via Pervaporation. J. Environ. Manag..

[11] Dmitrenko M.E., Penkova A.V., Kuzminova A.I., Morshed M., Larionov M.I., Alem H., Zolotarev A.A., Ermakov S.S., Roizard D. (2018). Investigation of New Modification Strategies for PVA Membranes to Improve Their Dehydration Properties by Pervaporation. Appl. Surf. Sci..

[12] Ogorodnikov S.K., Lesteva T., Kogan V., Zvukov D. (1971). Azeotropnye Smesi: Spravochnik (Azeotropic Mixtures: A Handbook).

[13] Benzaqui M., Semino R., Carn F., Tavares S.R., Menguy N., Giménez-Marqués M., Bellido E., Horcajada P., Berthelot T., Kuzminova A.I. (2019). Covalent and Selective Grafting of Polyethylene Glycol Brushes at the Surface of ZIF-8 for the Processing of Membranes for Pervaporation. ACS Sustain. Chem. Eng..

[14] Toti U.S., Aminabhavi T.M. (2002). Pervaporation Separation of Water-Isopropyl Alcohol Mixtures with Blend Membranes of Sodium Alginate and Poly(Acrylamide)-Grafted Guar Gum. J. Appl. Polym. Sci..

[15] Sajjan A.M., Jeevan Kumar B.K., Kittur A.A., Kariduraganavar M.Y. (2013). Novel Approach for the Development of Pervaporation Membranes Using Sodium Alginate and Chitosan-Wrapped Multiwalled Carbon Nanotubes for the Dehydration of Isopropanol. J. Membr. Sci..

[16] Dmitrenko M., Liamin V., Lahderanta E., Ermakov S., Penkova A. (2021). Mixed Matrix Membranes Based on Sodium Alginate Modified by Fullerene Derivatives with L-Amino Acids for Pervaporation Isopropanol Dehydration. J. Mater. Sci..

[17] Kuzminova A.I., Dmitrenko M.E., Poloneeva D.Y., Selyutin A.A., Mazur A.S., Emeline A.V., Mikhailovskii V.Y., Solovyev N.D., Ermakov S.S., Penkova A.V. (2021). Sustainable Composite Pervaporation Membranes Based on Sodium Alginate Modified by Metal Organic Frameworks for Dehydration of Isopropanol. J. Membr. Sci..

[18] Chaudhari S., Baek M., Kwon Y., Shon M., Nam S., Park Y. (2019). Surface-Modified Halloysite Nanotube-Embedded Polyvinyl Alcohol/Polyvinyl Amine Blended Membranes for Pervaporation Dehydration of Water/Isopropanol Mixtures. Appl. Surf. Sci..

[19] Rao K.S.V.K., Subha M.C.S., Sairam M., Mallikarjuna N.N., Aminabhavi T.M. (2007). Blend Membranes of Chitosan and Poly(Vinyl Alcohol) in Pervaporation Dehydration of Isopropanol and Tetrahydrofuran. J. Appl. Polym. Sci..

[20] Zhang W., Li G., Fang Y., Wang X. (2007). Maleic Anhydride Surface-Modification of Crosslinked Chitosan Membrane and Its Pervaporation Performance. J. Membr. Sci..

[21] Ge J., Cui Y., Yan Y., Jiang W. (2000). The Effect of Structure on Pervaporation of Chitosan Membrane. J. Membr. Sci..

[22] Tang S., Dong Z., Zhu X., Zhao Q. (2019). A Poly(Ionic Liquid) Complex Membrane for Pervaporation Dehydration of Acidic Water-Isopropanol Mixtures. J. Membr. Sci..

[23] Vedula S.S., Yadav G.D. (2021). Chitosan-Based Membranes Preparation and Applications: Challenges and Opportunities. J. Indian Chem. Soc..

[24] Castro-Muñoz R., González-Valdez J. (2019). New Trends in Biopolymer-Based Membranes for Pervaporation. Molecules.

[25] Zielińska K., Kujawski W., Chostenko A.G. (2011). Chitosan Hydrogel Membranes for Pervaporative Dehydration of Alcohols. Sep. Purif. Technol..

[26] Dmitrenko M., Zolotarev A., Plisko T., Burts K., Liamin V., Bildyukevich A., Ermakov S., Penkova A. (2020). Effect of the Formation of Ultrathin Selective Layers on the Structure and Performance of Thin-Film Composite Chitosan/PAN Membranes for Pervaporation Dehydration. Membranes.

[27] Zhang S., Zou Y., Wei T., Mu C., Liu X., Tong Z. (2017). Pervaporation Dehydration of Binary and Ternary Mixtures of N-Butyl Acetate, n-Butanol and Water Using PVA-CS Blended Membranes. Sep. Purif. Technol..

[28] Cao S., Shi Y., Chen G. (1999). Properties and Pervaporation Characteristics of Chitosan-Poly(N-Vinyl-2-Pyrrolidone) Blend Membranes for MeOH-MTBE. J. Appl. Polym. Sci..

[29] Yang D., Li J., Jiang Z., Lu L., Chen X. (2009). Chitosan/TiO_2_ Nanocomposite Pervaporation Membranes for Ethanol Dehydration. Chem. Eng. Sci..

[30] Chen J.H., Liu Q.L., Fang J., Zhu A.M., Zhang Q.G. (2007). Composite Hybrid Membrane of Chitosan–Silica in Pervaporation Separation of MeOH/DMC Mixtures. J. Colloid Interface Sci..

[31] Hung W.-S., Chang S.-M., Lecaros R.L.G., Ji Y.-L., An Q.-F., Hu C.-C., Lee K.-R., Lai J.-Y. (2017). Fabrication of Hydrothermally Reduced Graphene Oxide/Chitosan Composite Membranes with a Lamellar Structure on Methanol Dehydration. Carbon.

[32] Qiu S., Wu L., Shi G., Zhang L., Chen H., Gao C. (2010). Preparation and Pervaporation Property of Chitosan Membrane with Functionalized Multiwalled Carbon Nanotubes. Ind. Eng. Chem. Res..

[33] Vinu M., Senthil Raja D., Jiang Y.-C., Liu T.-Y., Xie Y.-Y., Lin Y.-F., Yang C.-C., Lin C.-H., Alshehri S.M., Ahamad T. (2018). Effects of Structural Crystallinity and Defects in Microporous Al-MOF Filled Chitosan Mixed Matrix Membranes for Pervaporation of Water/Ethanol Mixtures. J. Taiwan Inst. Chem. Eng..

[34] Harutyunyan L.R., Harutyunyan R.S., Gabrielyan G.A., Lasareva E.V. (2019). Modification of Chitosan and Chitosan Succinate by Surfactants and Investigation of Their Properties. Colloids Surf. A Physicochem. Eng. Asp..

[35] Lusiana R.A., Sangkota V.D.A., Santosa S.J. (2018). Chitosan Succinate/PVA-PEG Membrane: Preparation, Characterization and Permeation Ability Test on Creatinine. J. Kim. Sains Appl..

[36] Kochan J., Wintgens T., Wong J.E., Melin T. (2010). Properties of Polyethersulfone Ultrafiltration Membranes Modified by Polyelectrolytes. Desalination.

[37] Wang Q., Lu T.-D., Yan X.-Y., Zhao L.-L., Yin H., Xiong X.-X., Zhou R., Sun S.-P. (2020). Designing Nanofiltration Hollow Fiber Membranes Based on Dynamic Deposition Technology. J. Membr. Sci..

[38] Anantharaman A., Chun Y., Hua T., Chew J.W., Wang R. (2020). Pre-Deposited Dynamic Membrane Filtration—A Review. Water Res..

[39] Prihatiningtyas I., Van der Bruggen B. (2020). Nanocomposite Pervaporation Membrane for Desalination. Chem. Eng. Res. Des..

[40] Dmitrenko M.E., Penkova A.V., Kuzminova A.I., Atta R.R., Zolotarev A.A., Mazur A.S., Vezo O.S., Lahderanta E., Markelov D.A., Ermakov S.S. (2019). Development and Investigation of Novel Polyphenylene Isophthalamide Pervaporation Membranes Modified with Various Fullerene Derivatives. Sep. Purif. Technol..

[41] Liu Y., Tong Z., Zhu H., Zhao X., Du J., Zhang B. (2022). Polyamide Composite Membranes Sandwiched with Modified Carbon Nanotubes for High Throughput Pervaporation Desalination of Hypersaline Solutions. J. Membr. Sci..

[42] Msahel A., Galiano F., Pilloni M., Russo F., Hafiane A., Castro-Muñoz R., Kumar V.B., Gedanken A., Ennas G., Porat Z. (2021). Exploring the Effect of Iron Metal-Organic Framework Particles in Polylactic Acid Membranes for the Azeotropic Separation of Organic/Organic Mixtures by Pervaporation. Membranes.

[43] Dudek G., Turczyn R., Gnus M., Konieczny K. (2018). Pervaporative Dehydration of Ethanol/Water Mixture through Hybrid Alginate Membranes with Ferroferic Oxide Nanoparticles. Sep. Purif. Technol..

[44] Dudek G., Krasowska M., Turczyn R., Strzelewicz A., Djurado D., Pouget S. (2019). Clustering Analysis for Pervaporation Performance Assessment of Alginate Hybrid Membranes in Dehydration of Ethanol. Chem. Eng. Res. Des..

[45] Ren Y., Li T., Zhang W., Wang S., Shi M., Shan C., Zhang W., Guan X., Lv L., Hua M. (2019). MIL-PVDF Blend Ultrafiltration Membranes with Ultrahigh MOF Loading for Simultaneous Adsorption and Catalytic Oxidation of Methylene Blue. J. Hazard. Mater..

[46] Abdullah N., Rahman M.A., Dzarfan Othman M.H., Jaafar J., Aziz A.A. (2018). Preparation, Characterizations and Performance Evaluations of Alumina Hollow Fiber Membrane Incorporated with UiO-66 Particles for Humic Acid Removal. J. Membr. Sci..

[47] Burts K.S., Plisko T.V., Prozorovich V.G., Melnikova G.B., Ivanets A.I., Bildyukevich A.V. (2022). Development and Study of PVA–SiO2/Poly(AN-Co-MA) Dynamic Nanocomposite Membranes for Ethanol Dehydration via Pervaporation. Membr. Membr. Technol..

[48] Kuzminova A., Dmitrenko M., Zolotarev A., Korniak A., Poloneeva D., Selyutin A., Emeline A., Yushkin A., Foster A., Budd P. (2021). Novel Mixed Matrix Membranes Based on Polymer of Intrinsic Microporosity PIM-1 Modified with Metal-Organic Frameworks for Removal of Heavy Metal Ions and Food Dyes by Nanofiltration. Membranes.

[49] Gascón V., Jiménez M.B., Blanco R.M., Sanchez-Sanchez M. (2018). Semi-Crystalline Fe-BTC MOF Material as an Efficient Support for Enzyme Immobilization. Catal. Today.

[50] Shi J., Hei S., Liu H., Fu Y., Zhang F., Zhong Y., Zhu W. (2013). Synthesis of MIL-100(Fe) at Low Temperature and Atmospheric Pressure. J. Chem..

[51] Fu Y.-Y., Yang C.-X., Yan X.-P. (2013). Metal-Organic Framework MIL-100(Fe) as the Stationary Phase for Both Normal-Phase and Reverse-Phase High Performance Liquid Chromatography. J. Chromatogr. A.

[52] Kuzminova A., Dmitrenko M., Mazur A., Ermakov S., Penkova A. (2021). Novel Pervaporation Membranes Based on Biopolymer Sodium Alginate Modified by FeBTC for Isopropanol Dehydration. Sustainability.

[53] Hu X., Lou X., Li C., Ning Y., Liao Y., Chen Q., Mananga E.S., Shen M., Hu B. (2016). Facile Synthesis of the Basolite F300-like Nanoscale Fe-BTC Framework and Its Lithium Storage Properties. RSC Adv..

[54] Kujawski J., Rozicka A., Bryjak M., Kujawski W. (2014). Pervaporative Removal of Acetone, Butanol and Ethanol from Binary and Multicomponent Aqueous Mixtures. Sep. Purif. Technol..

[55] Eren M.Ş.A., Arslanoğlu H., Çiftçi H. (2020). Production of Microporous Cu-Doped BTC (Cu-BTC) Metal-Organic Framework Composite Materials, Superior Adsorbents for the Removal of Methylene Blue (Basic Blue 9). J. Environ. Chem. Eng..

[56] Akbari A., Karimi-Sabet J., Ghoreishi S.M. (2020). Intensification of Helium Separation from CH4 and N2 by Size-Reduced Cu-BTC Particles in Matrimid Matrix. Sep. Purif. Technol..

[57] Burts K.S., Plisko T.V., Bildyukevich A.V., Li G., Kujawa J., Kujawski W. (2022). Development of Dynamic PVA/PAN Membranes for Pervaporation: Correlation between Kinetics of Gel Layer Formation, Preparation Conditions, and Separation Performance. Chem. Eng. Res. Des..

[58] Kulkarni A.S., Sajjan A.M., Khan T.M.Y., Badruddin I.A., Kamangar S., Banapurmath N.R., Ayachit N.H., Ashwini M., Sharanappa A. (2021). Development and Characterization of Biocompatible Membranes from Natural Chitosan and Gelatin for Pervaporative Separation of Water–Isopropanol Mixture. Polymers.

[59] Achari D., Rachipudi P., Naik S., Karuppannan R., Kariduraganavar M. (2019). Polyelectrolyte Complex Membranes Made of Chitosan—PSSAMA for Pervaporation Separation of Industrially Important Azeotropic Mixtures. J. Ind. Eng. Chem..

[60] Zhang X., Zhang M.-X., Ding H., Yang H., Ma X.-H., Xu X.-R., Xu Z.-L., Tang C.Y. (2019). Double-Crosslinked GO Interlayer Framework as a Pervaporation Hybrid Membrane with High Performance. ACS Omega.

[61] Chanachai A., Jiraratananon R., Uttapap D., Moon G.Y., Anderson W.A., Huang R.Y.M. (2000). Pervaporation with Chitosan/Hydroxyethylcellulose (CS/HEC) Blended Membranes. J. Membr. Sci..

[62] Tsai H.A., Chen H.C., Chou W.L., Lee K.R., Yang M.C., Lai J.Y. (2004). Pervaporation of Water/Alcohol Mixtures through Chitosan/Cellulose Acetate Composite Hollow-Fiber Membranes. J. Appl. Polym. Sci..

[63] Kittur A.A., Kulkarni S.S., Aralaguppi M.I., Kariduraganavar M.Y. (2005). Preparation and Characterization of Novel Pervaporation Membranes for the Separation of Water–Isopropanol Mixtures Using Chitosan and NaY Zeolite. J. Membr. Sci..

[64] Jegal J., Lee K.-H. (1999). Chitosan Membranes Crosslinked with Sulfosuccinic Acid for the Pervaporation Separation of Water/Alcohol Mixtures. J. Appl. Polym. Sci..

[65] Anjali Devi D., Smitha B., Sridhar S., Aminabhavi T.M. (2005). Pervaporation Separation of Isopropanol/Water Mixtures through Crosslinked Chitosan Membranes. J. Membr. Sci..

[66] Ahmad A.L., Nawawi M.G.M., So L.K. (2005). Development of Novel NH _4_ Y Zeolite--Filled Chitosan Membranes for the Dehydration of Water--Isopropanol Mixture Using Pervaporation. Sep. Sci. Technol..

[67] Svang-Ariyaskul A., Huang R.Y.M., Douglas P.L., Pal R., Feng X., Chen P., Liu L. (2006). Blended Chitosan and Polyvinyl Alcohol Membranes for the Pervaporation Dehydration of Isopropanol. J. Membr. Sci..

[68] Liu Y.-L., Yu C.-H., Lee K.-R., Lai J.-Y. (2007). Chitosan/Poly(Tetrafluoroethylene) Composite Membranes Using in Pervaporation Dehydration Processes. J. Membr. Sci..

[69] Choudhari S., Kittur A., Kulkarni S., Kariduraganavar M. (2007). Development of Novel Blocked Diisocyanate Crosslinked Chitosan Membranes for Pervaporation Separation of Water–Isopropanol Mixtures. J. Membr. Sci..

[70] Veerapur R.S., Gudasi K.B., Aminabhavi T.M. (2007). Pervaporation Dehydration of Isopropanol Using Blend Membranes of Chitosan and Hydroxypropyl Cellulose. J. Membr. Sci..

[71] Liu Y.-L., Yu C.-H., Ma L.-C., Lin G.-C., Tsai H.-A., Lai J.-Y. (2008). The Effects of Surface Modifications on Preparation and Pervaporation Dehydration Performance of Chitosan/Polysulfone Composite Hollow-Fiber Membranes. J. Membr. Sci..

[72] Choudhari S.K., Kariduraganavar M.Y. (2009). Development of Novel Composite Membranes Using Quaternized Chitosan and Na+-MMT Clay for the Pervaporation Dehydration of Isopropanol. J. Colloid Interface Sci..

[73] Rachipudi P.S., Kittur A.A., Choudhari S.K., Varghese J.G., Kariduraganavar M.Y. (2009). Development of Polyelectrolyte Complexes of Chitosan and Phosphotungstic Acid as Pervaporation Membranes for Dehydration of Isopropanol. Eur. Polym. J..

[74] Varghese J.G., Kittur A.A., Rachipudi P.S., Kariduraganavar M.Y. (2010). Synthesis, Characterization and Pervaporation Performance of Chitosan-g-Polyaniline Membranes for the Dehydration of Isopropanol. J. Membr. Sci..

